# ATM Regulates Insulin-Like Growth Factor 1-Secretory Clusterin (IGF-1-sCLU) Expression that Protects Cells against Senescence

**DOI:** 10.1371/journal.pone.0099983

**Published:** 2014-06-17

**Authors:** Xiuquan Luo, Masatoshi Suzuki, Shanaz A. Ghandhi, Sally A. Amundson, David A. Boothman

**Affiliations:** 1 Departments of Pharmacology and Radiation Oncology, Laboratory of Molecular Cell Stress Responses, Program in Cell Stress and Cancer Nanomedicine, Simmons Cancer Center, University of Texas Southwestern Medical Center, Dallas, Texas, United States of America; 2 Center for Radiological Research, Columbia University Medical Center, New York, New York, United States of America; Roswell Park Cancer Institute, United States of America

## Abstract

Downstream factors that regulate the decision between senescence and cell death have not been elucidated. Cells undergo senescence through three pathways, replicative senescence (RS), stress-induced premature senescence (SIPS) and oncogene-induced senescence. Recent studies suggest that the *ataxia telangiectasia* mutant (ATM) kinase is not only a key protein mediating cellular responses to DNA damage, but also regulates cellular senescence induced by telomere end exposure (in RS) or persistent DNA damage (in SIPS). Here, we show that expression of secretory clusterin (sCLU), a known pro-survival extracellular chaperone, is transcriptionally up-regulated during both RS and SIPS, but not in oncogene-induced senescence, consistent with a DNA damage-inducible mechanism. We demonstrate that ATM plays an important role in insulin-like growth factor 1 (IGF-1) expression, that in turn, regulates downstream sCLU induction during senescence. Loss of ATM activity, either by genomic mutation (ATM-deficient fibroblasts from an *ataxia telangiectasia* patient) or by administration of a chemical inhibitor (AAI, an inhibitor of ATM and ATR), blocks IGF-1-sCLU expression in senescent cells. Downstream, sCLU induction during senescence is mediated by IGF-1R/MAPK/Egr-1 signaling, identical to its induction after DNA damage. In contrast, administration of an IGF-1 inhibitor caused apoptosis of senescent cells. Thus, IGF-1 signaling is required for survival, whereas sCLU appears to protect cells from premature senescence, as IMR-90 cells with sCLU knockdown undergo senescence faster than control cells. Thus, the ATM-IGF-1-sCLU pathway protects cells from lethality and suspends senescence.

## Introduction

Senescence has long been considered an important tumor suppression mechanism. Cellular senescence is a terminal state in which cells undergo permanent growth arrest accompanied by morphological changes, e.g., an enlarged and flattened cell shape. Cells can undergo senescence through three separate pathways [1], [2]: (i) Replicative senescence (RS), induced through shortening of telomeres as a result of chromosome replication; (ii) Stress induced-premature senescence (SIPS), induced by cellular stress, such as elevated oxygen levels or cytotoxic agents causing extensive DNA damage; and (iii) over-expression or hyper-activation of oncogenes, such as Ras, c-myc, or BRAF, whose mechanisms of senescence induction are poorly understood. These senescence pathways result in cells with uncontrolled oncogene activation or persistent and extensive DNA damage that permanently arrest growth, and prevent carcinogenesis. While non-replicative, senescent cells are still metabolically active and express secretory factors that may significantly alter the cellular microenvironment. Characterization of this ‘senescence secretome’ [3], and more importantly, determining the roles of secretory proteins in carcinogenesis are areas of active research. Indeed, a few studies have shown that senescent fibroblasts can promote tumor growth through certain secreted protein factors [4], [5]. Thus, senescence is most likely beneficial to an organism when cells are young, but a liability to organs as an organism gets older [6].

*Ataxia telangiectasia* mutant (ATM) kinase is a major regulator of certain pathways of senescence. Cells undergoing RS show telomere shortening due to repetitive replication leading to uncapped telomeres that can be recognized as DNA double strand breaks (DSBs) by ATM. Activated ATM can, in turn, signal downstream effectors. For example, p53 and p21 can mediate permanent cell cycle arrest [7], [8], [9], [10], [11]. In addition, uncapped telomeres can also activate other DNA damage signaling kinases, such as ATM-related kinase (ATR) and Ku-dependent DNA protein kinase (DNA-PK). These kinases play redundant roles in RS for sensing and responding to the environment, as well as age-related damage accumulation. Unlike RS, the detailed mechanisms underlying SIPS are less understood. Evidence indicates that induction of SIPS is also strongly linked to DNA damage [10], [12], [13]. For example, most cell stressors that induce SIPS are DNA damage-inducing agents, such as growth in elevated oxygen, exposure to ionizing radiation (IR), and treatment with drugs that generate DSBs [14], [15], [16], [17], [18], [19]. All of these agents can activate ATM, which appears to be an important mediator of SIPS [15], [17]. Nevertheless, factors that regulate the intercellular decision-making steps of senescence (permanent growth-arrest) and survival of cells during the senescence process have not been elucidated.

Secretory clusterin (sCLU) is a stress-inducible, ∼80 kDa secreted glycoprotein implicated in various biological processes [20], including cellular senescence. Although sCLU over-expression during cellular senescence has been reported, and sCLU expression noted as a biomarker of senescence [19], [21], the exact mechanisms regulating its expression during aging have not been elucidated. One of sCLU’s primary functions is to clear cell debris from injured cells or tissues, thereby acting as an ‘extracellular chaperone’ that binds stressed, unfolded proteins for recycling [22], [23]. Additionally, sCLU can protect cells from apoptosis through its interaction with the pro-apoptotic protein, Bax [22]. sCLU also functions as a tumor promoting factor and is commonly over-expressed in multiple human cancers, including breast, colon and prostate. For example, sCLU over-expression has been linked to increased aggressiveness and metastatic ability in breast cancer [23], [24], and is used as a biomarker to detect triple-negative breast cancers [25]. Moreover, sCLU over-expression in various cancers results in resistance to various anti-cancer drugs and ionizing radiation (IR). Conversely, down-regulation of sCLU by antisense RNA or small interfering RNA (siRNA) knockdown enhances the radio−/chemo-sensitivities of human cancer cells [26].

In prior studies, we demonstrated that insulin-like growth factor 1 (IGF-1) induced sCLU via activation of the ATM/IGF-1/IGF-1R/Src/Erk1-2/Egr-1 pathway in response to DNA damaging agents, and we showed that sCLU is a sensitive measure of endogenous and exogenous genomic stress [27], [28]. Linking the known damage-inducible regulation of sCLU with prior reports of senescence-mediated sCLU over-expression, we hypothesized that ATM-mediated IGF-1 production during senescence regulated sCLU expression. Here, we investigated the regulation and function of sCLU during cellular senescence. We show that sCLU expression is regulated during senescence through the ATM-dependent production of IGF-1 during RS and SIPS, but not in oncogene-induced senescence. IGF-1 expression, in turn, stimulates the Src/MAPK pathway leading to significant sCLU expression. Importantly, we show that sCLU protects cells from senescence and that selective siRNA-mediated sCLU knockdown enhanced progression to senescence. In contrast, preventing IGF-1-IGF-1R signaling by blocking IGF-1R kinase activity not only suppressed sCLU expression, but caused significant lethality, specifically in senescent cells. Our data strongly suggest that the ATM-IGF-1/IGF-1R-sCLU pathway plays an overall protective role during senescence, whereby IGF-1 stimulated, IGF-1R downstream signaling is required for survival and sCLU expression suspends senescence. We speculate that accumulation of IGF-1 and sCLU in the media of an aging microenvironment of specific tissues could contribute to tumor promotion.

## Experimental Procedures

### Cell Lines and Reagents

IMR-90, BJ, HE49 cells (from Coriell) were cultured in MEM medium (Invitrogen, Carlsbad, CA, USA), plus 10% FBS, 2 mM glutamine, 0.5 mM sodium pyruvate. HBEC cells were a gift from Dr. John Minna (UT Southwestern Medical Center) and cultured in KSFM medium (Invitrogen) as described [29]. Primary human ATM-deficient AT fibroblasts, AT2052 and GM03487, were purchased from Coriell and cultured in MEM medium with 15% FBS, 2 mM glutamine, 0.5 mM sodium pyruvate. Immortalized AT fibroblasts [27], with or without restored ATM expression, were cultured in DMEM medium with 15% FBS, 2 mM glutamine. All cells were grown with nonessential amino acids. Antibodies against sCLU (B5), nCLU (H330), total Erk, phosphorylated Erk, p53 (DO1), β-actin, Egr-1, Src and phosphorylated Src were from Santa Cruz. Antibodies against IGF-1 receptor β-chain, phospho-IGF-1 receptor β-chain (Tyr1135/1136), p16 and p21 were from Cell Signaling. Anti-α-tubulin antibody was from Calbiochem. IGF-1 receptor inhibitor, AG1024 was purchased from Sigma. ATM inhibitor, AAI (CGK733) was purchased from Calbiochem.

An inducible sCLU shRNA plasmid, pTRIPZ-shCLU, was constructed by sub-cloning the shCLU containing fragment from pSM2C-shCLUmir vector (Open Biosystem, Lafayette, CO, USA) into the pTRIPZ empty vector (Open Biosystem). The shCLU containing fragment was amplified using a pair of primers, 5′-CTTCAGGTTAACCCAACAG-3′ and 5′-CGAAGTGATCTTCCGTCACAA-3′ from pSM2C-shCLUmir, and subcloned into the pTRIPZ vector using *Xho I* and *Mlu I*. The resulting plasmid, pTRIPZ-shCLU was sequenced for insert confirmation. The inducible non-silencing shRNA control plasmid, pTRIPZ-nonSilence was purchased from Open Biosystem. The CLU promoter fused to luciferase was previously described [28].

### Oncogene-induced Senescence

Retrovirus encoding constitutive active Ras^V12^ was transducted into young IMR-90 cells to induce senescence as described [30]. The same batch of IMR-90 cells was also transducted with retrovirus alone to serve as control.

### Western Blot Analysis

Cells were washed with PBS once, lysed with 50–100 µl lysis buffer (50 mM Tris pH 6.8, 1% SDS, 10% 2-mercaptoethanol, 1×protease inhibitor cocktail (Roche), 1×phosphatase inhibitor cocktail 1 (Sigma), 1×phosphatase inhibitor cocktail II (Sigma)). Protein concentrations were determined by the Bradford Method and Bio-Rad protein assay dye reagent (Bio-Rad). Western assays were performed as described [28].

### ELISA Assays

IGF-1 was detected using capturing and detecting antibodies, MAB291 and MAF291 (R&D Systems, Minneapolis, MN, USA) following the protocol provided by the company. Samples were normalized to cell number and the total volume of medium.

### Senescence-associated β-gal Staining

Cells were washed with PBS, fixed (2% formaldehyde, 0.2% glutaraldehyde in 1×PBS) for 5 mins, and immersed in stain solution (40 mM Sodium Citrate, pH 6.0, 150 mM NaCl, 5 mM potassium ferrocyanide, 5 mM potassium ferricyanide, 2 mM MgCl_2_, 1 mg/ml X-gal) and incubated overnight at 37°C.

### Statistics

Paired Student’s t-tests (n≥3) were used to analyze experiments, which were performed at least three times unless otherwise specified. Statistics of population doubling curves were calculated using mixed model analyses by Dr. Jingsheng Yan and Dr. Xian-Jian Xie in the Biostatistical Core of the Simmons Comprehensive Cancer Center, U T Southwestern Medical Center.

## Results

### Expression of Secretory Clusterin is Transcriptionally Increased during Senescence

**Figure 1:**
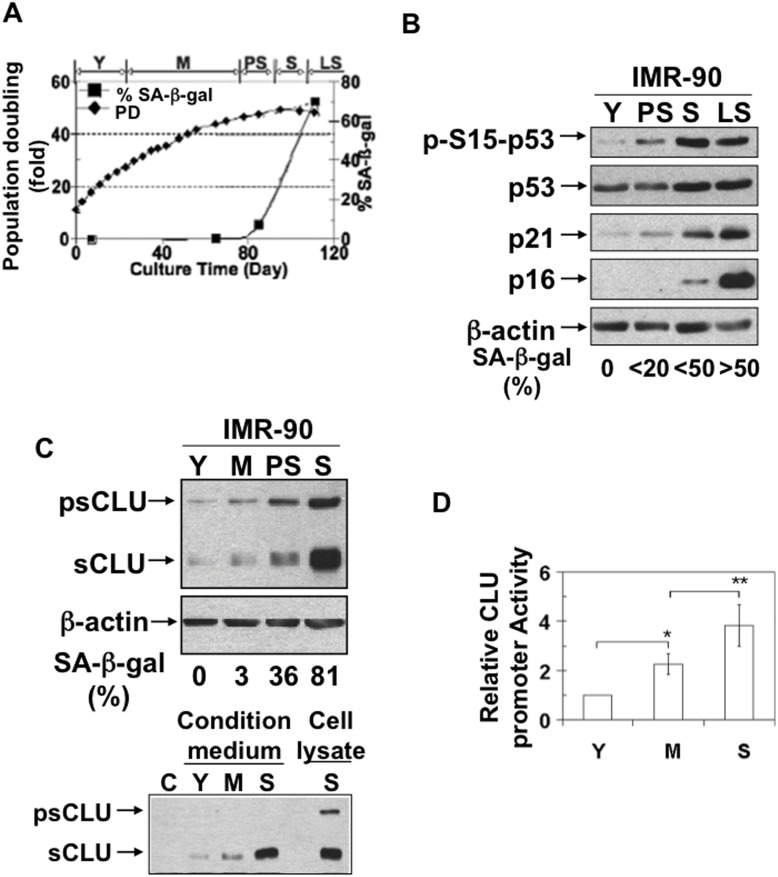
sCLU expression is increased during senescence. Young (Y), middle-age (M), premature-senescent (PS), senescent (S), and late senescent (LS) IMR-90 cells were generated by continuous culture. (A) The “age” of the IMR-90 cells was determined by population doublings and %SA-β-gal+ cell measurements. Experiments were repeated three or more times in triplicate each, and representative results are shown. (PD, population doubling). (B) Senescence markers, phospho-ser15-p53, total p53, p21 and p16 levels, were markedly increased during senescence. (C) Both the precursor (psCLU) and the mature secretory (sCLU) forms of clusterin were induced in total cell lysates during senescence. sCLU levels increased in the media of senescent IMR-90 cells. (D) The promoter activity of sCLU was increased during senescence. *, p-value≤0.05, **, p-value≤0.01.

To generate cells at different ages and stimulate stress-induced premature senescence (SIPS), human normal diploid IMR-90 lung fibroblasts were continually cultured under 20% O_2_. Young (Y), middle-age (M), premature-senescent (PS), senescent (S) and late senescent (LS) IMR-90 cells were generated over time, as indicated by decreased population doubling and increased expression of senescent-associated β-galactosidase-positive (SA-β-gal+) cells (Figure 1A). Senescence was further confirmed by monitoring specific biomarkers (i.e., known senescent mediators), including elevated levels of phosphorylated serine15 p53 (Ser^15^ p53), total p53, p21 and p16 proteins (Figure 1B). We also noted a dramatic increase in pre-mature (∼60 kDa psCLU) and mature (∼40 kDa sCLU) forms of clusterin (a.k.a. apolipoprotein J) protein during senescence (Figure 1C); mature sCLU is ∼80 kDa formed by dimerization of two ∼30 kDa α and β sCLU peptides that are heavily glycosylated, but appear as ∼40 kDa forms under SDS reducing conditions. Both psCLU and sCLU protein forms were concomitantly increased in middle-aged IMR-90 cells, and expression of both protein forms were further increased in premature-senescent (PS) cells, with peak levels demonstrated in senescent (S) IMR-90 cells. Mature sCLU levels were also found dramatically increased in the ‘conditioned’ media of middle-aged and senescent fibroblasts (Figure 1C). To explore whether CLU up-regulation was controlled at the transcriptional level, a human 4280 bp CLU promoter-luciferase reporter (hCLUp-Luc) was used. hCLUp-Luc activity was increased 2-fold in middle-aged IMR-90 cells compared to young cells, while 4-fold increased promoter activity was observed in senescent IMR-90 cells (Figure 1D). Overall, these data strongly suggested that sCLU protein expression was transcriptionally up-regulated in IMR-90 cells during SIPS.

### sCLU is Induced in Cells Undergoing RS or SIPS

**Figure 2:**
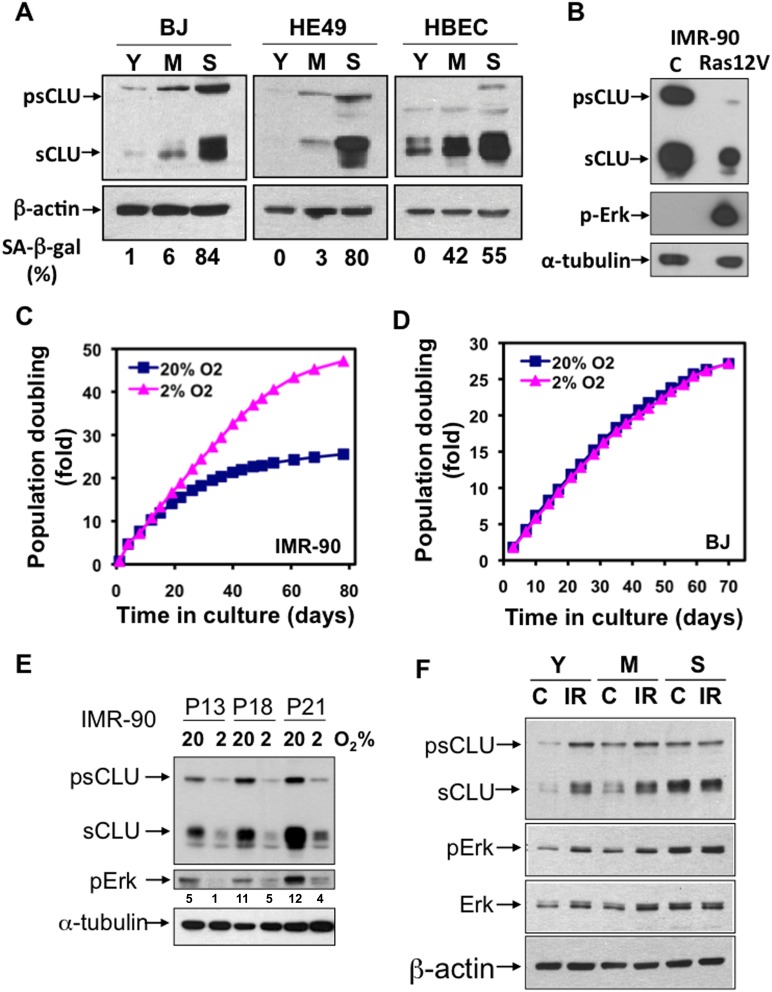
sCLU expression is differently regulated in SIPS, RS and oncogene-induced senescence. (A) sCLU levels were increased during senescence in BJ, HE45 and HBEC cells. BJ, HE45 and HBEC cells were aged by continuous cell culture and senescence status determined by %SA-β-gal+ cells. (B) Forced expression of Ras12V represses sCLU expression. Young IMR-90 cells were transduced with lentiviral-mediated Ras12V expression. After cells exhibited typical senescence morphology, stained SA-β-gal+, and were growth arrested, they were harvested and sCLU levels monitored by Western blotting. Phospho-Erk-1/2 was probed to monitor the effects of Ras12V over-expression. α-Tubulin was used for a loading control. (C) IMR-90 cells cultured in 20% oxygen (O_2_) underwent senescence faster than cells cultured in 2% O_2_. The population doubling of IMR-90 cells cultured in 20% O_2_ decreased faster than cells cultured in 2% O_2_. (D) BJ cells were not affected by different O_2_ tensions. In contrast to IMR-90 cells, the population doublings of BJ cells were identical whether cultured in 20% or 2% O_2_. (E) sCLU levels increased in IMR-90 cells cultured in 20% O_2_ compared to levels found in IMR-90 cells cultured in 2% O_2_ in both early and late passage cells. (F) Young (Y), middle-aged (M), and senescent (S) IMR-90 cells were treated with IR (10 Gy). psCLU, sCLU, total Erk-1/2 (Erk) and phosphorylated Erk-1/2 (pErk) were detected by western blot analyses. For (C) and (D), representative results are presented from at least three repeat experiments with similar results.

To investigate whether sCLU expression increases were specific to cells undergoing SIPS, we examined other normal human diploid cells, BJ, HE49 and human bronchial epithelial cells (HBECs), that undergo RS, and are not subject to SIPS under the relatively elevated O_2_ levels found under normal tissue culture growth conditions. In all three cells, psCLU and sCLU levels were increased in cells undergoing RS (Figure 2A). Thus, sCLU levels are enhanced by both RS and SIPS.

IMR-90 cells are hypersensitive, and significantly growth-arrested, by elevated O_2_ levels in cell culture [31]. IMR-90 cells underwent senescence much more rapidly when cultured in 20% O_2_ compared to cells cultured under 2% O_2_, as indicated by decreased population doubling times and dramatically increased levels of SA-β-gal+ cells (Figure 2C). In contrast, BJ cells are not particularly sensitive to oxygen (O_2_) and no significant difference in their population doubling times were noted in cells cultured under 20% versus 2% O_2_ (Figure 2D). Thus, BJ cells primarily undergo RS regardless of O_2_ levels, and sCLU induction responses were identical (as shown in Figure 2A) in these cells regardless of O_2_ levels.

To investigate the effect of O_2_ levels on sCLU expression during SIPS versus RS in IMR-90 cells, we examined psCLU and sCLU steady state levels in IMR-90 cells cultured under 20% or 2% O_2_ growth conditions. sCLU levels were significantly elevated in IMR-90 cells cultured under 20% O_2_ at passages (13–21) where cells were undergoing SIPS, compared to identical cells cultured in 2% O_2_ (Figure 2E), in which no significant senescence was noted (Figure 2C). Thus, sCLU expression correlated well with SIPS in IMR-90 cells. Finally, we examined cells undergoing oncogene-induced senescence by transfecting constitutive-active Ras12V into young IMR-90 cells, a method commonly used to induce oncogene-stimulated senescence [30]. Interestingly, basal sCLU expression was repressed in Ras12V-expressed young IMR-90 cells (Figure 2B). These results are consistent with prior findings suggesting that Ras over-expression negatively regulates sCLU expression [32], and strongly suggests that sCLU is not induced during oncogene-induced senescence.

Prior research from our laboratory demonstrated that sCLU induction in response to DNA damaging agents, such as ionizing radiation (IR), occurred through IGF-1 production, stimulating IGF-1R/MAPK/Erk-1/2 signaling, culminating in activated Egr-1 that transcriptionally regulated sCLU expression [28]. We previously demonstrated that such responses were mediated by ATM activation, and not through ATR [27]. Since both RS and SIPS activate ATM via cellular DNA damage responses (DDR), we treated young (P13, PD#17), middle-aged (P18, PD#36) and senescent (P21, PD#92) IMR-90 cells with IR, then examined sCLU expression. sCLU levels were dramatically increased in young cells in response to IR treatment, while no additionally increases in sCLU levels were noted in irradiated senescent IMR-90 cells, which expressed extremely elevated basal levels of this pro-survival protein compared to young IMR-90 cells (Figure 2F). These results indicated that senescence-induced sCLU might employ the same signaling pathway as DDR-induced sCLU [27]. Consistent with this pathway, we noted that Erk-1 was activated (increased p-Erk-1/t-Erk) during senescence (Figures 2E and 2F), and activation of Erk-1 was further induced after IR treatment in young (Y) or middle-aged (M) IMR-90 cells. In contrast, IR exposure of senescent (S) IMR-90 cells caused no further Erk-1 activation (Figure 2F). Thus, the signaling pathway required for sCLU expression was already saturated in senescent IMR-90 cells. These results again strongly suggested that senescence-induced sCLU expression is mediated through the same signaling pathway as DDR-induced sCLU expression noted in Goetz et al. [27].

### ATM Mediates IGF-1-sCLU Expression during Senescence

**Figure 3:**
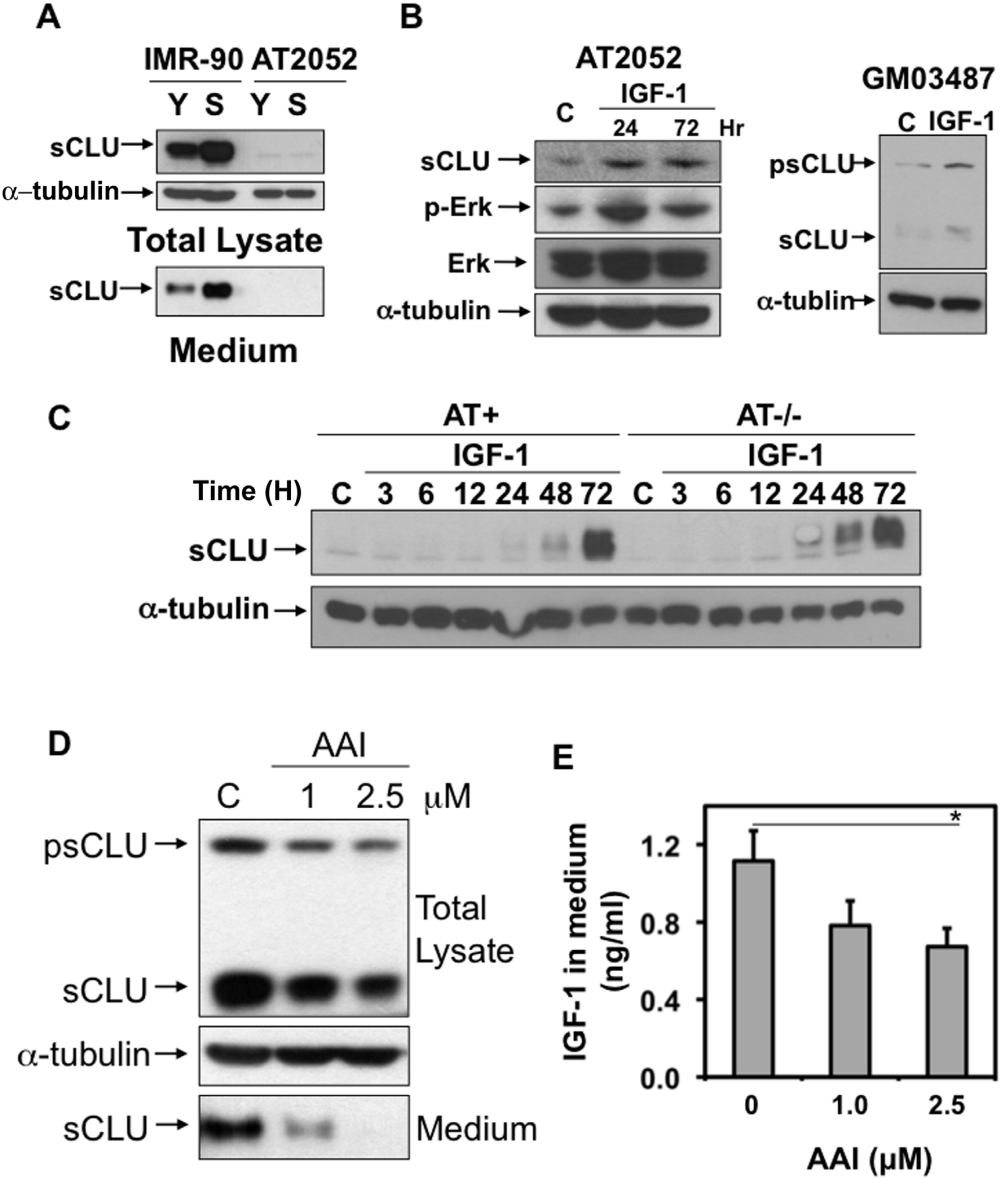
ATM is required for sCLU induction during senescence. (A) ATM-deficient AT cells have extremely low basal sCLU levels, where expression is regulated purely by IGF-1 in the medium [27]. sCLU levels were increased in both total lysates and in the medium from senescent IMR-90 cells, however, sCLU was not changed during senescence in AT2052 cells. The %SA-β-gal+ cells in the population from young IMR-90 cells was <5%, whereas the percentage of senescent IMR-90 cells was >60%. The %SA-β-gal+ cells in the Young AT2052 cell population was <20%, whereas the senescent population was >60% for AT2052 cells. (B) IGF-1 induced sCLU in ATM-deficient, AT fibroblasts. ATM-deficient AT2052 or GM03487 cells were cultured in 0.2% FBS medium overnight, and then treated with IGF-1 (10 ng/ml). Cells were harvested at the indicated times and sCLU as well as total and phospho-Erk-1/2 levels were measured by Western Blotting from total cell lysates. α-Tubulin levels were used for loading. (C) IGF-1 induces sCLU in genetically matched ATM-deficient and -proficient AT cells. Immortalized AT cells (AT−/−) and wild type, ATM reconstituted cells (ATM+) were cultured in 0.2% FBS medium overnight. IGF-1 (10 ng/ml) was then added in both ATM+ and ATM−/− cells. Cells were harvested at indicated times and sCLU and α-tubulin levels were monitored as described above. (D) Inhibition of ATM activity represses sCLU expression in senescent IMR-90 cells. psCLU and sCLU were repressed by the ATM inhibitor, AAI, in both total cell lysates and medium from senescent IMR-90 cells (%SA-β-gal+ were >60%). These cells were pre-cultured in 0.2% FBS medium overnight, then treated with 1.0 or 2.5 µM AAI or exposed to vehicle alone. After treatment (48 h), cells and medium were collected and Western Blot analyses were performed to measure sCLU level. α-Tubulin levels were used to monitor loading. (E) Inhibition of ATM activity repressed IGF-1 expression in senescent IMR-90 cells. IGF-1 levels in the media from cells in (D) were measured using ELISAs as described in ‘Materials and Methods’. Human recombinant IGF-1 was used as standard. Results were normalized to both total volume of medium and total cell number.

ATM plays a central role in DDRs, as well as cellular senescence through RS or SIPS, to signal downstream effectors controlling survival and cell death [10]. To test whether ATM regulated sCLU expression during cellular senescence, we aged human diploid *Ataxia telangiectasia* (AT) patient (AT2052) fibroblasts that lack functional ATM expression. AT2052 cells expressed significantly lower basal level expression of sCLU compared to wild-type ATM+ IMR-90 cells. These data are consistent with prior data from our lab, where ATM controlled IGF-1-sCLU expression after DNA damaging agents, and low basal levels of sCLU noted in AT cells were noted as resulting from detectable IGF-1 in the growth medium [27]. As expected, sCLU was not induced in senescent AT2052 versus IMR-90 cells (Figure 3A). Furthermore, AT cells were capable of inducing sCLU, since exposure of AT2052 cells with IGF-1 resulted in significant sCLU expression in 24 h compared to untreated control cells, and sCLU levels remain elevated 72 h after IGF-1 exposure (Figure 3B). Accordingly, activated Erk (increased P-Erk/t-Erk) was concomitantly noted 24 h after IGF-1 treatment (Figure 3B). These results indicated that signaling from IGF-1/IGF-1R to sCLU was still intact in ATM-deficient AT2052 cells. To further confirm this finding, another AT cell line (GM03487) was treated with IGF-1, and similarly, sCLU expression was increased in GM03487 cells 24 h post-treatment (Figure 3B). Similar results were also observed using a pair of immortalized AT cells lacking or reconstituted with ATM (Figure 3C), as demonstrated in Goetz et al., [27]. Collectively, these data are consistent with an ATM-mediated, IGF-1-regulated sCLU expression pathway that signals through the IGF-1 receptor (IGF-1R)/MAPK/Erk-1/2/Egr-1 signaling pathway. The low level of constitutive sCLU expression, and lack of induction of sCLU during senescence in AT cells is due to the lack of functional ATM and not due to signaling from IGF-1/IGF-1R to sCLU expression. These data are consistent with the hypothesis that ATM controls IGF-1 expression during senescence, as it does after DDRs [27].

To further demonstrate a role for ATM in sCLU expression in senescent IMR-90 cells, the ATM and ATR inhibitor (AAI in 0.2% FBS medium) was used to decrease intracellular psCLU and sCLU expression, as well as mature sCLU expression in conditioned medium (Figure 3D). We further analyzed IGF-1 levels in the medium and found that inhibition of ATM significantly decreased IGF-1 expression in these cells (Figure 3E). Taken together, these results suggested that ATM was an important mediator of sCLU expression during senescence, where it regulates sCLU expression by controlling IGF-1 levels.

### sCLU Induction during Senescence is Mediated by IGF-1R/MAPK/Erk-1/2/Egr-1 Signaling

**Figure 4:**
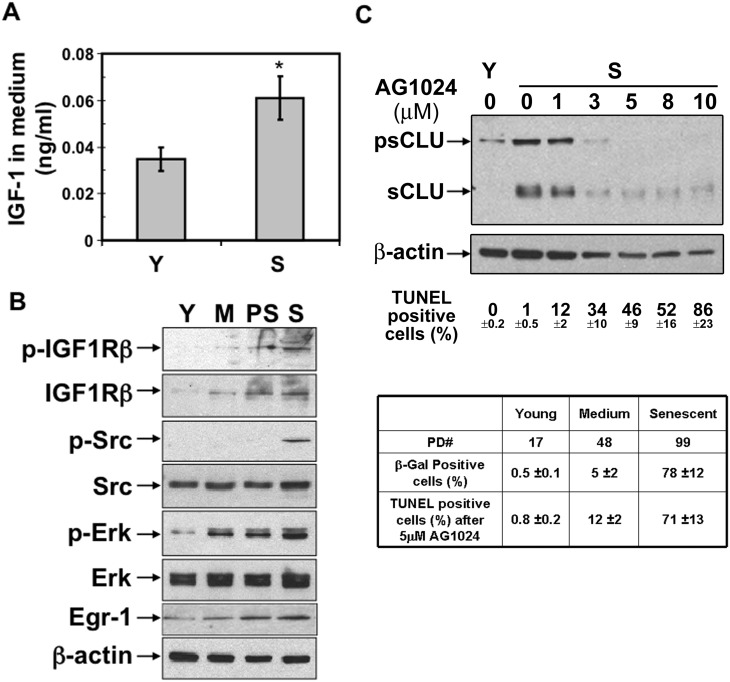
sCLU induction during senescence is mediated through IGF-1R/MAPK/Erk/Egr-1 signaling pathway. (A) IGF-1 expression was increased in senescent IMR-90 cells. IGF-1 levels in media from young or senescent IMR-90 cells were measured using ELISAs as described above, and normalized to media volume and adjusted cell number (4×10^4^ cells). (B) Total IGF-1 receptor (IGF-1R), phosphorylated IGF-1 receptor (p-IGF-1R), Src, phosphorylated Src, Erk-1/2, phosphorylated-Erk-1/2, and Egr-1 levels in young (Y), middle-age (M), premature-senescent (PS), and senescent (S) IMR-90 cells were determined by western blot analyses. β-Actin levels were measured to monitor loading. (C) Exposure to the indicated doses of the IGF-1R tyrosine kinase inhibitor (AG1024) repressed sCLU levels in senescent IMR-90 cells. psCLU and sCLU were detected by western blot analysis. β-Actin levels were monitored to show equal loading. Senescent IMR-90 cells exposed to varying doses of AG1024 (µM as indicated for 24 h) showed dose-dependent increases in apoptosis (monitored by TUNEL + staining). Only senescent, and not young or middle-aged IMR-90, cells were hypersensitive to AG1024. In the experiment outlined below the western blot analyses cells were exposed to AG1024 (5 µM) for 48 h and therefore higher levels of apoptotic cells were noted in senescent IMR-90 cells compared to cells exposed for 24 h above. Note that only senescent cells were hypersensitive to these inhibitor treatments. All experiments were repeated three times in triplicate.

Our data strongly suggested that functional ATM could regulate IGF-1 expression during RS or SIPS pathways. Indeed, IGF-1 expression was approximately two-fold higher in conditioned media from senescent compared to young IMR-90 cells (Figure 4A). Analyses of differential signal transduction responses in senescent versus young IMR-90 cells showed that IGF-1 receptor (IGF-1R) levels were activated (elevated P-IGF-1R/t-IGF-1R) in senescent cells; antibodies to human IGF-1R are not ideal. Src and Erk-1/2 kinase levels were also elevated in middle-aged and senescent compared to young IMR-90 cells. The transcription factor, Egr-1, which binds the hCLU promoter and mediates sCLU expression [28], was significantly increased during senescence in IMR-90 cells (Figure 4B). To further confirm the specificity of IGF-1/IGF-1R/MAPK/ERK-1/2/Egr-1 signaling in sCLU induction during senescence, we treated senescent IMR-90 cells with the IGF-1R inhibitor, AG1024, which effectively blocked IR-induced sCLU expression [27], [28] and also suppressed sCLU expression in senescent IMR-90 cells in a dose-dependent manner (Figure 4C). While significantly suppressing sCLU expression, we also noted that senescent IMR-90 cells exposed to the IGF-1R inhibitor (AG1024) underwent significant programmed cell death (apoptotic) responses in a dose-dependent manner (Figure 4C). Interestingly, young and middle-aged cells did not respond to AG1024 in the same manner with no significant apoptosis noted after exposure to 5 µM of the drug (Figure 4C). Collectively, these results strongly suggest that when cell undergo RS or SIPS, but not oncogene-induced senescence, ATM-dependent DDR leads to increased IGF-1 expression that stimulates IGF-1R/MAPK/Erk-1/2/Egr-1 signaling that leads to concomitant elevated sCLU levels. The data further suggest that the IGF-1/IGF-1R-sCLU pathway is required for the overall survival of senescent cells, whereby suppressing this pathway induces an intracellular death decision.

### sCLU Knockdown Promotes Cellular Senescence

**Figure 5:**
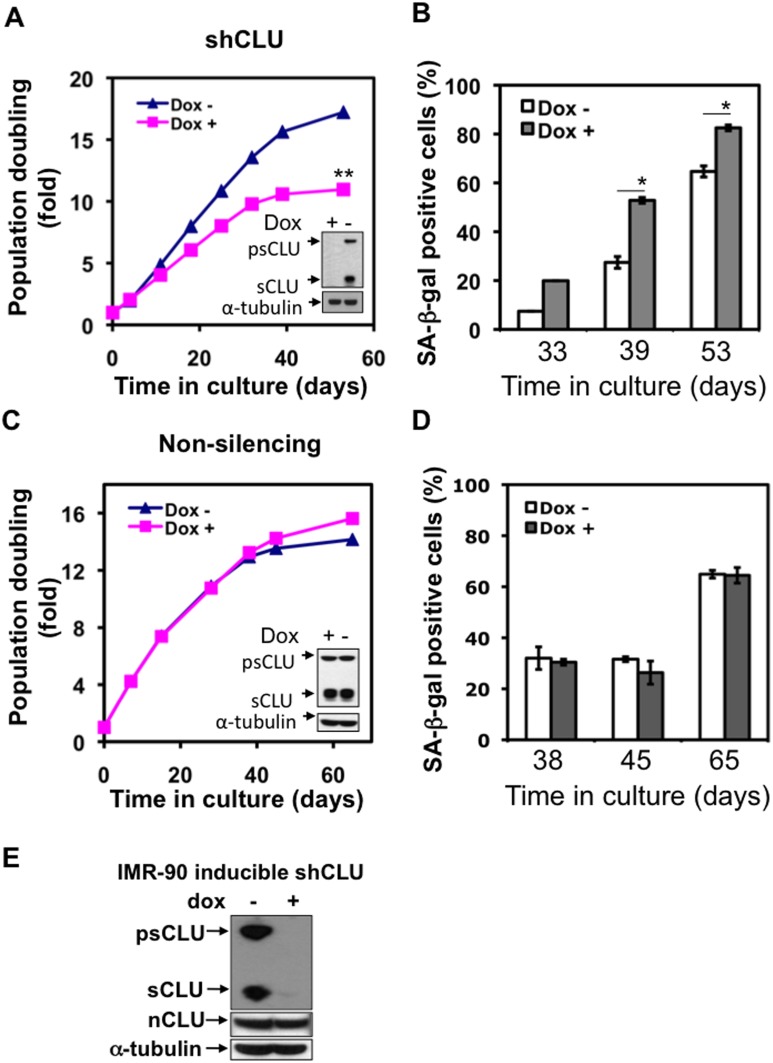
sCLU protects cells from senescence. (A) sCLU was knocked down in an IMR-90 pooled population containing a lentiviral-mediated inducible shRNA-sCLU construct cultured in presence of doxycycline (dox), while expression of sCLU was not affect in the same pooled population in the absence of doxycycline (dox). The population doubling of IMR-90 cells with inducible shRNA-sCLU decreased faster when cultured in the presence of dox compared to in cells cultured without dox. **, p-value of two curves was ≤0.01. Experiments were repeated three times, starting from infection of young IMR-90 cells with selection of a pTRIPZ-shCLU-containing pooled population. Similar results were observed in all three experiments and representative results from one experiment are shown. (B) IMR-90 cells with sCLU knockdown had significantly higher %SA-β-gal+ cells after being cultured for 33, 39, and 53 days with or without dox. SA-β-gal+ staining was performed to identify senescent cells. *, p-value ≤0.05. (C) Controls included a pooled IMR-90 cell population contain an inducible shRNA-non-targeted, scrambled shRNA construct were cultured in presence or absence of dox. Growth rates and sCLU levels were not affected in the shRNA-Scr (non-targeted) IMR-90 cell populations. Population doubling of IMR-90 cells with inducible non-targeting shRNA was not different when cultured with/without doxycycline. The experiments were repeated three times similarly as inducible shCLU experiments. Representative results of three experiments were shown here. (D) After culturing for 38, 45, and 65 days with or without dox, SA-β-gal+ staining was performed to identify senescent cells in IMR-90 pooled populations with inducible shRNA-Scr (non-targeting) vector. (E) Specificity of shCLU knockdown in conditional IMR-90 senescent cells. IMR-90 pooled populations containing the pTRIPZ-shCLU vector was cultured with or without doxcycline (100 nM for 3 days) and sCLU and nCLU levels were monitored using Western blot analyses. α-Tubulin levels were shown as a loading control. Note that sCLU and not nCLU levels were specifically knocked down (>90%) in cells exposed to dox. In these experiments, IMR-90 cells were cultured in 95% air-5% CO_2_, estimated at ∼20% O_2_.

sCLU functions as an extracellular chaperone, protecting cells from inflammation, and suppressing apoptosis by its proposed interaction with Bax [22], [33]. Its role in senescence has not been defined. Given the increased apoptotic responses in cells deficient of sCLU expression (Figure 4C) exposed to an IGF-1R inhibitor, we theorized that the effects of the IGF-1R inhibitor were due to the proposed apoptotic inhibitory effects of psCLU/sCLU. To explore sCLU’s role in senescence, we generated a conditionally inducible, doxycycline-regulated shRNA-sCLU knockdown lentiviral expression vector, pTRIPZ-shCLU. When infected into cells, pTRIPZ-shCLU controlled expression of shRNA specific to the intron I/Intron III junction found only in sCLU mRNA, and not in nuclear clusterin (nCLU) mRNA (Figure 5E); nCLU is a cytosolic form of clusterin that induces cell death when activated and translocated to the nucleus [35], [36]. Early passage IMR-90 cells were first transduced with lentiviral pTRIPZ-shCLU, then selected with puromycin to generate a pooled population. pTRIPZ-shCLU-containing IMR-90 cells were then cultured with or without Dox, and population doublings (PDs) and %SA-β-gal+ cells monitored over time. Interestingly, Dox-treated, pTRIPZ-shCLU-containing IMR-90 cells with continuous sCLU knockdown showed decreased population doublings at ∼passage 20 (Figure 5A), suggesting that loss of sCLU expression enhanced SIPS responses in IMR-90 cells. Significant population doubling time differences were noted (p-value of two population doubling curves = 0.0001), with control cells plateauing at ∼40 days in culture, while Dox-treated IMR-90 cells underwent senescence at day 32 (Figure 5A). Enhanced senescence was confirmed by significant differences in the %SA-β-gal+ cells, where at least two-fold increases were noted compared to untreated pTRIPZ-shCLU IMR-90 cells that expressed sCLU at day 39 (Figure 5B). sCLU-shRNA knockdown was confirmed in Dox-exposed pTRIPZ-shCLU IMR-90 cells by Western blotting, while sCLU levels in cells similarly cultured without Dox were not affected and were significantly elevated (Figure 5A). To verify that these results were not affected by addition of Dox or lentiviral infection alone, we generated pooled clones using the same vector, but contain a non-targeting shRNA (pTRIPZ-non silencing). As shown in Figures 5C and 5D, Dox-inducible non-silencing shRNA had no affect on sCLU expression (Figure 5C), both population doubling times and %SA-β-gal+ cells were not significantly different with or without Dox exposure over the life of the IMR-90 cultures (Figure 5C). These results strongly suggested that decreased sCLU expression due specifically to shRNA-sCLU knockdown, facilitated senescence in IMR-90 cells. Thus, sCLU appears to play a protective role from senescence in IMR-90 cells. Interestingly, however, loss of sCLU did not lead to significant induction of apoptosis, strongly suggesting that the role of sCLU in senescence is to suspend the process and not to keep these cells alive. These data also strongly suggest that IGF-1/IGF-1R signaling, in contrast, is a major contributing factor that protects against programmed cell death induction in cells.

## Discussion

**Figure 6:**
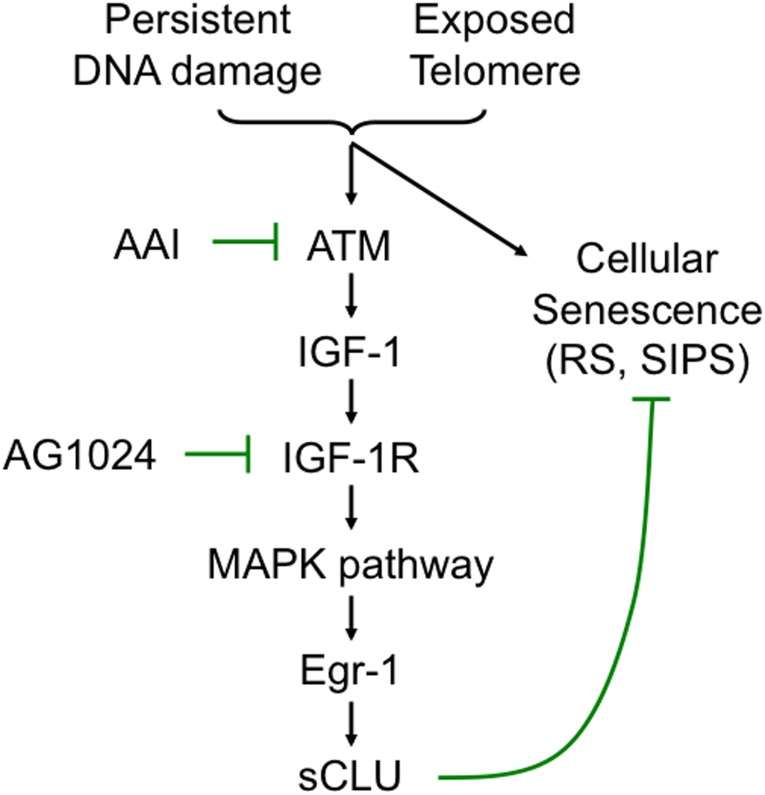
A model of the signaling pathway that mediates sCLU induction during senescence showing the role of sCLU in senescence compared to IGF-1/IGF-1R signaling required to prevent apoptosis. AAI and AG1024 are small molecule chemical inhibitors that inhibit ATM and ATR, and IGF-1 tyrosine kinase receptor (IGF-1R), respectively.

Here, we show that sCLU is up-regulated at a transcription level during both RS and SIPS, but is not elevated in response to oncogene-induced senescence. This is consistent with prior findings in the literature showing that sCLU was induced in WI-38 cells during RS or after H_2_O_2_-induced SIPS [19], [21]. Mechanistically, we also demonstrated that sCLU was induced during senescence through the ATM/IGF-1/IGF-1R/MAPK/Erk-1/2/Egr-1 signaling pathway, a pathway that is also stimulated and regulates sCLU induction during DDR (Figure 6). These results support the observation that cellular senescence processes share significant overlap with DDR pathways. And many key factors (e.g., ATM, p53, p21) that are involved in the DDR in human cells also play important roles in cellular senescence responses.

Importantly, our data clearly support a role for ATM in the induction of sCLU levels in senescent cells by controlling IGF-1 expression. Upon DNA damage, including uncapped telomere ends of extensive DNA lesions created in SIPS responses, ATM is recruited to the damage site and activated via phosphorylation [34]. Activated ATM, in turn, stimulates several proteins that can mediate DNA damage repair, cause transient growth arrest, and possibly lead to cell death or senescence. Ironically, loss of ATM function sensitizes cells to DNA damage, leading to enhanced cell death and possibly senescence. ATM can be activated by naked telomeres and trigger cellular senescence signaling during RS [7], [11], [35]. Similarly, ATM activation by overwhelming DSB lesions may also lead to senescence signaling during SIPS. However, ATM also plays a protective role in aging and cellular senescence, since ATM-deficient AT cells undergo senescence faster [38], [39], [40]. Consistently, AT patients also age faster. Our finding that ATM up-regulates and controls the IGF-1-sCLU expression axis [27], as well as sCLU protecting cells from senescence, may help to explain the role of ATM in senescence/aging protection (Figure 6).

Our results demonstrate that sCLU protects cells from senescence, suggesting that a role of increased sCLU expression in senescent cells is to counteract senescence by some as yet known mechanism. This result is consistent with the role of ATM in protecting cells from senescence. AT fibroblasts expressed low basal levels of sCLU, and failed to induce sCLU during senescence or DDR responses, due to the lack of ATM signaling (Figure 3A) that controls IGF-1 production [27]. Thus, the role of sCLU expression in DDR responses mirrors ATM, as both AT cells and cells knocked down for sCLU expression are hypersensitive to oxidative stress and senescence. AT patients also age faster [38], [39], [40], and patients with lowered circulatory sCLU levels also age less well than Centenarians in a recent study [36]. Re-introduction of ATM into AT cells increased the lifespan of AT cells and restored sCLU induction responses after DDR or senescence induction pathways [27]. Collectively, these results suggest that sCLU may play a role(s) in aging. Furthermore, increased levels of sCLU may protect cells from senescence (Figure 6).

Our original hypothesis was that as an anti-apoptotic protein, sCLU may increase the population doubling by protecting cells from spontaneous apoptosis. However, when sCLU was knocked down in middle-aged or senescent IMR-90 cells, we did not observe an increase in apoptosis in this cell population. These data suggest that the anti-apoptotic function of sCLU may somehow not be operative during senescence and may not contribute to increased cell growth. Surprisingly, Dox-induced shRNA-sCLU knockdown IMR-90 cells showed increased SA-β-gal+ stained cells, confirming its role in senescence protection (Figure 5). sCLU also functions as an extracellular chaperone, that helps clear unfolded proteins from the surrounding microenvironment (conditioned medium). Several studies have shown that other chaperones, like heat shock proteins (HSPs), were involved in cellular senescence [37], [38], [39], [40], [41]. Decreasing or inhibiting Hsp90 or Hsp72 induced cellular senescence, while over-expression of Hsp72 suppressed senescence pathways [37], [38], [39]. A small heat shock protein, Hsp27, also suppressed senescence by modulating p53 signaling [40]. Thus, sCLU may protect cells from senescence by decreasing cellular stress caused by the accumulation of denatured proteins, since both the cellular level of HSPs and protein quality control declines during senescence [41]. Furthermore, other findings, as well as our results have shown that extracellular sCLU can interact with several cell surface receptors, such as IGF-1R, TGF-β receptor I and TGF-β receptor II, and its interactions with these receptors modulates their activities in as yet undefined mechanisms. Both IGF-1 and TGF-β receptor-mediated signaling pathways are involved in cellular senescence, thus sCLU might modulate (i.e., prevent) senescence by regulating the activities of these receptors. It should be noted that knocking down sCLU expression alone does not alter, or may enhance, IGF-1/IGF-1R signaling [27].

Finally, our data strongly indicate that the remaining IGF-1/IGF-1R signaling pathway, not affected by shRNA-sCLU specific knockdown, but inhibited by IGF-1R receptor kinase-specific agents (e.g., AG1024) are essential for the survival of senescent cells and cause these cells to undergo programmed cell death (Figure 4C). The potential significance of these differential responses clinically could well depend on the age of a patient. Since senescence is probably tumor-suppressive in young individuals, but potentially tumor-promoting in aged individuals, efficacious adjuvant treatment of patients using sCLU knockdown therapies (using OGX011), or exposure to IGF-1R tyrosine kinase inhibitors, may well depend on the overall age of the individual. In the future, physicians may well decide to use OGX-011 in a young patient to promote the tumor-suppressive properties of this therapy to cause onset (and possibly increased overall numbers) of senescence cells. Then, later in life and if IGF-1R inhibitors are truly specific for senescent cells, the physician may subscribe such inhibitors for prevention of senescence-induced microenvironmental changes that might promote tumor survival and progression. Further mechanism studies and trials in young and aged mice in vivo will be required to explore these interesting senescence-induced micro-environmental dynamics.
